# Diabetic ketoacidosis among patients admitted to a general hospital in Ethiopia: a spatial analysis

**DOI:** 10.1093/eurpub/ckac129.649

**Published:** 2022-10-25

**Authors:** E Paiola, A Sartorello, G Andreani, A Tsegaye, S Tardivo, F Manenti, R Benoni

**Affiliations:** 1 Department of Diagnostics and Public Health, University of Verona, Verona, Italy; 2 Doctors with Africa CUAMM, Padua, Italy

## Abstract

Non-Communicable diseases are rapidly increasing in low- and middle-income countries. The number of patients with diabetes is estimated to reach 4.7 million in Ethiopia by 2045. Ensuring access to care is critical to improving the management and clinical outcome of diabetic patients. The study describes the characteristics of patients with diabetic ketoacidosis (DKA) and evaluates the relationship between the severity of clinical presentation and the travel time to the hospital. A retrospective cohort study was conducted on the charts of patients admitted for DKA at St. Luke Catholic Hospital (SLCH), Wolisso, Oromia Region (Ethiopia), between 01/01/2021 and 31/08/2021. Demographic and clinical data were collected. Negative binomial regression was used to explore the relationship between the incidence of admissions for DKA and travel time to the hospital. Logistic regression was used to estimate the odds of insulin treatment. Results were presented with 95% confidence intervals. During the study period, 651 patients were admitted, including 77 (11.8%) for DKA (33 females (42.9%) and 44 males (57.1%)), with no differences based on diabetes type (p = 0.258). The mean age was 35 years (IQR 19.0-52.0). Mean BMI was 18.4 kg/m2 (IQR 15.6-19.5), with no differences based on diabetes type (p = 0.639). Cumulative incidence of hospitalizations was significantly correlated to travel time to the hospital (p = 0.039) with an Incident Rate Ratio of 1.01%[1.00-1.02]. The cumulative incidence ranged from 7.0%[4.5-10.3] in Wolisso to 30.8%[14.3-51.8] in Ameya, the most distant district. The relative probability of insulin treatment was higher with increasing time to SLCH (OR 1.11[1.02-1.21] p = 0.027). The hospitalization rate for DKA was significantly correlated with the travel time to the hospital. Access to care is therefore a key factor for health that should be taken into account by improvement programs and the spatial analysis of travel time could help focus on priority areas.

**Key messages:**

• Spatial analysis can be a robust tool to tailor population healthcare needs on its own topography.

• Health policies must consider that accessibility can influence the severity of clinical presentation.

